# Pre-Exposure Prophylaxis (PrEP) for HIV Infection in Cisgender and Transgender Women in the U.S.: A Narrative Review of the Literature

**DOI:** 10.1007/s10508-020-01903-8

**Published:** 2021-06-01

**Authors:** Aleta Baldwin, Brenda Light, Waridibo E. Allison

**Affiliations:** 1 Department of Public Health, California State University, Sacramento, Solano Hall 3014, 6000 J Street, Sacramento, CA 95819 USA; 2 School of Nursing, University of Texas at Austin, Austin, TX USA; 3 Division of Infectious Disease, Department of Medicine, University of Texas Health Science Center at San Antonio, San Antonio, TX USA

**Keywords:** HIV prevention, Pre-exposure prophylaxis, Gender, PrEP, Cisgender women, Transgender women

## Abstract

Using a socioecological approach, this review describes the peer-reviewed literature on oral pre-exposure prophylaxis (PrEP) among both cisgender (cis women) and transgender women (trans women) in the U.S. A search of the PubMed database and HIV-related conference abstracts generated over 2,200 articles and abstracts. Of these, 103 fulfilled review inclusion criteria. Most of the existing research presents findings on individual-level factors associated with PrEP use such as willingness and perceived barriers. There was far less investigation of factors related to PrEP at more distal ecological levels. Though trans women are at greater risk of HIV infection than cisgender women, less is known about this population group with respect to PrEP despite their inclusion in many major clinical trials. Further, the literature is characterized by a persistent conflation of sex and gender which makes it difficult to accurately assess the reviewed research on HIV prevention and PrEP apart from risk group. Informed by these findings, we highlight specific opportunities to improve access to PrEP and reduce socioecological barriers to PrEP care engagement for cisgender and transgender women.

## Introduction

According to the CDC (2017), women, presumably cisgender women (those assigned female sex at birth who identify as women), accounted for at least 19% of HIV diagnoses in the U.S. in 2017. Transgender women (those assigned male sex at birth who identify as women) have disproportionately high rates of HIV infection (Poteat, Reisner, & Radix, 2014), and the risk of HIV infection among transgender women in the U.S. is 34 times greater than the general population (Baral et al., 2013). It is unclear what proportion of new HIV diagnoses in the U.S. occur in transgender women as HIV surveillance data on this population are limited (CDC, 2019).

Pre-exposure prophylaxis (PrEP) is an evidence-based intervention to prevent HIV infection utilizing chemoprophylaxis (Grant et al., 2010; Mujugira et al., 2011). In 2012, Truvada, a fixed dose combination of emtricitabine/tenofovir was approved by the US Food and Drug Administration (FDA) for use as oral PrEP medication. Despite its effectiveness, PrEP is not widely used by transgender or cisgender women at the highest risk of infection (Wilson et al., 2015; Wu et al., 2016). Of nearly 80,000 unique individuals starting PrEP in the U.S. between 2012 and 2015, fewer than a quarter were women and the proportion of women accessing PrEP has steadily decreased since 2012 (Mera et al., 2016). The PrEP-to-need ratio, a standardized measure of new PrEP users relative to HIV diagnoses, is five times lower among females compared to males, a figure that indicates unmet need in HIV prevention such as PrEP (Siegler et al., 2018).

A second oral PrEP medication, Descovy, a fixed dose combination of emtricitabine/tenofovir alafenamide, received FDA approval in October 2019. Importantly, cisgender women were not included in the clinical trials for Descovy and it is not FDA approved to protect against HIV transmission during vaginal sex. Consequently, this second PrEP option is unlikely to benefit cisgender women or contribute to increased PrEP access for them in the short-term. Cabotegravir, a long-acting injectable form of PrEP, has been shown in preliminary studies to be more effective than Truvada in preventing HIV in high-risk men and transgender women (Landovitz et al., 2020). The most recently reported findings in November 2020 have shown Cabotegravir to be highly effective for HIV prevention for cisgender women; these findings led to the Drug Safety and Monitoring Board recommendation to stop the blinded phase of the trial early (WHO, 2020). A long-acting injectable PrEP formulation will be important to improve PrEP uptake and adherence for those who prefer not to take a daily pill or have difficulty doing so including difficulty related to sexual partner disapproval or pressure.

Most PrEP research samples are constructed and described according to risk categories (e.g., Men who have sex with men: “MSM,” People who use drugs: “PWUD”). This increases the difficulty of assessing potential differences between groups of people in the same risk category. For example, grouping transgender women with cisgender MSM prevents a full understanding of trans women’s experiences of the PrEP care continuum. It also prevents a synthesis of cisgender and transgender women’s experiences of the PrEP care continuum. In contrast to much of the existing research and associated literature reviews on PrEP in our populations of interest (Bailey, Molino, Vega, & Badowski, 2017; Escudero et al., 2015; Koechlin et al., 2017; Sheth, Rolle, & Gandhi, 2016), this review is focused on gender rather than on risk category. Thus, we present what is known about PrEP in both cisgender (henceforth “cis”) and transgender (henceforth “trans”) women in the U.S.

As with HIV risk factors, barriers and facilitators to PrEP arise from multiple social and biological factors (Baral et al., 2013; Poteat et al., 2015). Socioecological models of health (SMH) are widely used to describe the multiple levels of influence on health and to document interactions between factors at multiple levels to better assess risk and guide prevention efforts. Such models are useful in organizing reviews of the literature (Dulin et al., 2018; Stangl, Lloyd, Brady, Holland, & Baral, 2013). This study relies on a SMH to organize and present relevant findings on PrEP in cis and trans women by socioecological level.

## Method

The review was conducted beginning in March 2018. The terms ((pre-exposure prophylaxis)) OR ((antiretroviral prophylaxis)) OR ((pre-exposure chemoprophylaxis)) OR ((PrEP)) AND ((HIV)) OR ((AIDS)) were searched in the PubMed database. Given the relatively limited literature in the area of focus, we utilized broad inclusion criteria. To be eligible for inclusion, articles had to be English language and published within time frame from January 1, 2005, to December 31, 2017. Exclusion criteria were (1) no cis or trans women in the study sample, (2) studies without primary data such as reviews, editorials and commentaries, (3) studies conducted entirely outside of the U.S., (4) studies of non-oral PrEP, (5) studies focused on pharmacokinetics and/or pharmacodynamics with no clinical implications, and (6) studies with no distinction made between genders of participants.

**Fig. 1:**
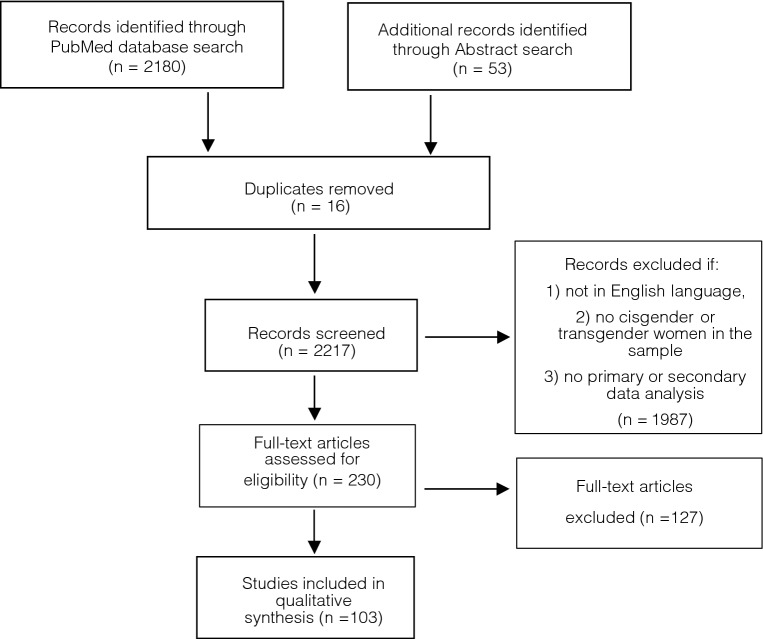
Modified PRISMA Flow Diagram of studies included in systematic review

Following the initial search, the titles and abstracts of all results were reviewed and excluded according to the exclusion criteria by author BL. Full-text articles were reviewed by two faculty authors (AB and WA). Additionally, conference abstracts for major HIV-related conferences were searched from 2008 to 2017 as online abstracts were not available prior to 2008. The conferences were: ID Week, which has existed since 2012 as the combined meeting annual meeting of the infectious Disease Society of America/Society for Healthcare Epidemiology of America/HIV Medical Association/Pediatric Infectious Disease Society; the annual Conference on Retroviruses and Opportunistic Infections; and the biennial International AIDS Society conference. Where there was disagreement about whether to exclude an article or abstract, disagreement was resolved by discussion between authors AB and WA. The article selection process is depicted in Fig. 1, with a modified Preferred Reporting Items for Systematic Reviews and Meta-Analyses (PRISMA) flow diagram (Moher, Liberati, Tetzlaff, Altman, & The PRISMA Group, 2009).

This review utilizes a socioecological model to organize research findings. All records meeting inclusion criteria were entered into a results matrix collating the following information from each paper: study sample, methodology, setting, and findings at each of the five ecological levels: individual, interpersonal, community, institutional (organization), and policy/societal levels. Though many factors described are crosscutting, and reflect more than one level of influence, guided by the model described by McElroy et al. (1988), we operationalized the levels as follows. At the individual level, we included findings related to characteristics of individuals, such as awareness of and attitudes toward PrEP, as well as perceived and actual behavioral risk. At the interpersonal level, we included findings related to relationships between women and their close social networks, including their sexual and romantic partners, peers, and family. At the community level, we included findings related to identity communities, including group norms as well as findings related to structural communities, for instance resources in their geographical area. At the institutional or organizational level, we included findings related to larger social institutions such as medicine, education, and the media. Conceiving of healthcare providers as institutional/organization actors, we also included findings related to experiences in health care and interactions between women and their healthcare providers at this level. Finally, research findings related to broader social forces, including laws or policies that distribute resources, were included at the societal/policy level. Relevant findings from included studies were initially entered into the matrix by one of four student researchers. Each record was reviewed and checked for accuracy by author AB, who then completed the matrix coming to a consensus about any uncertainty with matrix accuracy with author WA. The literature on trans women and cis women was reviewed separately first and then combined for synthesis.

## Results

### Data Extraction and Management

The initial database search yielded 2,180 records. Further identified were 53 conference abstracts. Sixteen duplicate records were removed and so were 1987 articles and abstracts that, prima facie, did not meet eligibility requirements. We therefore assessed 230 full-text records for inclusion. Of these, 106 articles and abstracts fulfilled inclusion criteria and were eligible for review. Two articles that technically met inclusion criteria were removed post hoc because they were studies of healthcare providers with no findings directly about PrEP in cis or trans women. A single case study was also excluded, and thus, a total of 103 articles and abstracts were included.

### General Characteristics of Included Studies

The characteristics of included studies are presented in Table 1. Most studies utilized a quantitative methodology (77.7%, *n* = 80), and the majority of study locations were in the northeastern region of U.S. Table 2 shows characteristics of included study samples with regard to sex and gender. Similar proportions of study samples were comprised of either (1) cis men and cis women (32.0%, *n* = 33) or (2) cis men and trans women (28.1%, *n* = 29). In just over half of the included studies, we found at least one instance of sex and gender conflation, for example sex (e.g., male and female) and gender (e.g., men, women) used interchangeably or male and female being referred to as genders (53.4%, *n* = 55). In 9.7% of studies, we found at least once instance of sexuality and gender conflation, for example transgender being referred to as a sexual identity, or MSM utilized as a gender category (*n* = 10). In 44.8% of studies of cis men and trans women (*n* = 13), the sample included less than 5% trans women. In over a quarter of these studies, trans women were misgendered and referred to as “men” (27.6%, *n* = 8). Of the studies with mixed-gender samples (e.g., cisgender men and transgender women; cisgender men and cisgender women), 32.3% (*n* = 20) presented or discussed no findings specific to PrEP in cisgender or transgender women and, as such, met all the inclusion criteria but contained no relevant data to extract (Table 3 provides a list of these sources).

**Table 1:** Characteristics of included studies

	%	*N* = 103
*Study location*		
Northeast		65
South		52
West		34
Midwest		22
USA (Nationally representative or convenience samples)		12
Puerto Rico		3
*Design and method*		
Quantitative	77.7	80
Qualitative	17.4	18
Mixed methods	4.9	5

Studies with multiple locations prevent assessment of proportion of study locations

**Table 2:** Sex- and gender-related characteristics of included studies

		**%**	*N* = 103
*Study Sample*			
Cisgender men and women/male and female		32.0	33
Cisgender men and transgender women (MSM/TGW)		28.1	29
Cisgender women/female		19.4	20
Both cis and trans participants^a^		10.7	11
Trans women and gender non-binary participants		5.8	6
Other (e.g., clinicians, stakeholders)		3.9	4
*Studies Conflating*			
Sex and gender		53.4	55
Sexuality and gender		9.9	10
*Studies with Combined Samples of Cisgender Men and Transgender Women*		28.1	29
With less than 5% trans women participants		44.8	13
Reporting no findings specific to trans women beyond frequency in sample		72.4	21
Misgendering trans women (refer to them as “men” or “MSM”)		27.6	8

^a^Excludes combined samples of cisgender men and transgender women

**Table 3:** Studies with no findings specific to transgender or cisgender women (*n* = 20)

First author, Year	Sex/Gender of Participants (Percent cis women and/or trans or gender non-binary participants)
Amico et al. (2014)	Cisgender men and transgender women (14%)
Amico et al. (2016)	Cisgender men and transgender women (11%)
Eaton et al. (2017a)	Cisgender men and transgender women (1.5%)
Ellorin et al. (2015)	Cisgender men and transgender women (1%)
Gandhi et al. (2016)	Cisgender men and transgender women (11%)
Golub et al. (2013)	Cisgender men and transgender women (3.8%)
Golub (2014)	Cisgender men and transgender women (3.5%)
Golub et al. (2017)	Cisgender men and transgender women/gender non-binary individuals (4.5%)
Grant et al. (2010)	Cisgender men and transgender women (1%)
Gulick et al. (2017)	Cisgender men and transgender women (2%)
Kellerman et al. (2005)	Cisgender men, cisgender women (7%), and transgender individuals (3%)
Knopf et al. (2017)	Cisgender men and transgender women/gender non-binary individuals (10.3%)
Lalley-Chareczko et al. (2017)	Cisgender men and transgender women (10%)
Mannheimer et al. (2015)	Cisgender men and transgender women (1.7%)
Moore et al. (2017)	Cisgender men and transgender women (0.7%)
Mulligan et al. (2015)	Cisgender men and transgender women (10%)
Shrestha et al. (2017a, d)	Cisgender men and cisgender women (41%)
Wall et al. (2016)	Cisgender men, cisgender women (11%), and transgender individuals (6%)
Wenzel et al. (2017)	Cisgender men, cisgender women (27.8%), and transgender women (1%)

## Individual Level Findings

### Awareness

Common across the reviewed literature, in a range of environments and populations, was low awareness of PrEP. Multiple studies comparing cis men and women found lower PrEP awareness in women (Farhat, Greene, Paige, Koblin, & Frye, 2017; Koper et al., 2015; Misra & Udeagu, 2017), including among those living with HIV (Jayakumaran, Aaron, Gracely, Schriver, & Szep, 2016). Depending upon sample characteristics and geographical region, awareness ranged between 0% (Auerbach, Banyan, & Riordan, 2012) and 33% (Peitzmeier et al., 2017). In a sample of mostly black women from across the U.S., awareness was less than 10% (Auerbach, Kinsky, Brown, & Charles, 2015), but was around 26% in both a small sample of black and Latina women in New York City (Collier, Colarossi, & Sanders, 2017) and residents of a women’s shelter in Miami, Florida (Doblecki-Lewis et al., 2016). The highest reported awareness among cis women was in those who did sex work (33%), including those who were also classified as women who inject drugs (WWID) (Peitzmeier et al., 2017). Among WWID, those who reported sex work were three times more likely to be aware of PrEP than those who did not (Walters et al., 2017). Nevertheless, low awareness held in WWID (Metz et al., 2017; Walters, Rivera, et al., 2017).

Awareness of PrEP was similarly low among trans women (Sevelius, Keatley, Calma, & Arnold, 2016b; Wilson, Jin, Liu, & Raymond, 2015). Among young trans women in large metropolitan areas, awareness of PrEP was just over 30% (Kuhns et al., 2016). The lowest reported PrEP awareness among trans women was 13.7%, but was higher among those with an HIV-positive partner (Wilson et al., 2015). In a more recent qualitative study of trans women, knowledge of PrEP was relatively high at 64% (Wood, Lee, Barg, Castillo, & Dowshen, 2017). An additional study found that PrEP awareness did not differ significantly by gender among a sample of black cisgender men and transgender women (Eaton et al., 2017b).

### Acceptability, Willingness, and Intention

Acceptability of PrEP was generally high among black cis women, including those who were sampled as part of an adolescent–parent dyad (Bond & Gunn, 2016; Shah, Gillespie, Holt, Morris, & Camacho-Gonzalez, 2016). Knowledge of the limited side effects associated with PrEP facilitated interest in cis women (Collier et al., 2017), as did experiencing past condom failure (Flash et al., 2014a, b). Short-term worry about HIV infection was not associated with PrEP acceptability (Garfinkel, Alexander, McDonald-Mosley, Willie, & Decker, 2017). Interest in PrEP was high among trans women, with one study finding nearly 70% being “somewhat” or “very” interested in PrEP (Kuhns et al., 2016).

Willingness to use PrEP was lower among cis women compared to men in most studies with mixed-cisgender samples (Kwakwa et al., 2016; Kwakwa, Gaye, & Bessias, 2014; Whiteside, Harris, Scanlon, Clarkson, & Duffus, 2011), but there were important differences between groups of women documented as well. In all but one study (Kwakwa, Gaye, & Bessias, 2014), black cis women were more willing to use PrEP than their white counterparts (Garfinkel et al., 2017; Willie, Kershaw, Campbell, & Alexander, 2017; Wingood et al., 2013). Willingness also appeared to be associated with younger age (Doblecki-Lewis et al., 2016; Garfinkel et al., 2017) and a history of sex work (Peitzmeier et al., 2017). There were no significant differences in willingness to use PrEP related to gender in people who use drugs (PWUD) (Shrestha, Altice, Karki, & Copenhaver, 2017b), among whom effectiveness was the most important criterion in determining whether to take PrEP (Kuo et al., 2016; Metz et al., 2017; Stein, Thurmond, & Bailey, 2014).

Intention to use PrEP was higher among black cis women compared with their white counterparts, among those with less than high school education compared to college graduates, and among unemployed women compared to those employed full-time (Dunkle, Wingood, Camp, & DiClemente, 2008). While interest in PrEP was high among trans women (Kuhns et al., 2016), this may not be the case among trans women with indications for PrEP; one study found that no trans women participants who were candidates for PrEP were actually willing to take it (Wilson et al., 2015). Consistent with the findings on PrEP uptake and adherence, the biggest influence on openness to PrEP among trans women was the need to prioritize hormone therapy (Sevelius et al., 2016b).

### Uptake, Adherence, and Discontinuation

Three-quarters of all 624,000 heterosexually active adults in the U.S. with indications for PrEP are women (Smith et al., 2015), though women, specifically black women, are less likely to be screened for PrEP indications compared to other groups (Elopre, Kudroff, Westfall, Overton, & Mugavero, 2017; Okoye, Chang, Weissman, & Duffus, 2017). Relatedly, PrEP uptake among cis women was low overall in the studies reviewed (Scott et al., 2018; Wu et al., 2016), though it varied by region and population (Laufer, O’Connell, Feldman, & Zucker, 2015), ranging from less than 15% (Mayer, Levine, Grasso & Gelman 2015; Montgomery et al., 2016; Patel et al., 2017) to 42% (Flash et al., 2014b) of samples. Adherence to PrEP was lower and discontinuation was higher among cis women compared with cis men (Blackstock, Patel, Felsen, Park, & Jain, 2017; Marcus et al., 2016; Ohl et al., 2017). PrEP was found to become ineffective for cis women after fewer missed doses compared to cis men and trans women due to lower bioavailability in vaginal mucosa compared to anal mucosa (Cottrell et al., 2016).

PrEP use was also lower in trans women compared to cis men, despite numerous documented PrEP indications among trans women (Kuhns et al., 2016), but these differences were not always significant when tested (Cohen et al., 2015; Eaton et al., 2017b). In most of the studies reviewed, PrEP uptake among trans women was between 2.5% (Clement et al., 2017) and 9% of study samples (Belkind et al. 2016). And, while trans women were as likely as cis men to remain engaged within the first year of initiating PrEP (Glidden et al., 2015; Liu et al., 2014; Liu et al., 2016), multiple studies found that adherence to PrEP was lower among those trans women taking hormone therapy compared to both trans women and cis men who were not (Deutsch et al., 2015; Grant et al., 2014). In two studies, depression was associated with lower adherence to PrEP in trans women (Defechereux et al., 2016; Mehrotra et al., 2016).

### Perceived Risk

There were notable differences between cis women and men with respect to risk perception, with women perceiving themselves to be at lower risk for HIV (Koper et al. 2015; Kwakwa et al., 2016). However, cis women appear to be less likely to underestimate their actual HIV risk than men (Kwakwa et al., 2014a, b). Monogamous black cis women were found to have low perceived risk despite partners’ infidelity or knowing a person who was HIV positive (Flash et al., 2012).

### Barriers to PrEP Use

Among the numerous barriers to PrEP use reported in the literature were stigma associated with utilizing HIV prevention methods (Collier et al., 2017; Goparaju et al., 2017), potential side effects including interactions with contraception (Auerbach et al., 2012; Auerbach et al., 2015; Smith, Toledo, Smith, Adams, & Rothenberg, 2012), efficacy (Auerbach et al., 2012; Smith et al., 2012), frequency of medical visits (Collier et al., 2017), and the difficulty associated with a daily medication in addition to condom use (Khawcharoenporn, Kendrick, & Smith, 2012; Smith et al., 2012). In one study with a mixed-cisgender sample from a high HIV prevalence geographical area, there were few differences between men and women with respect to these barriers to PrEP use (Koper et al., 2015). Among a group of postnatal cis women using PrEP, 50% reported adherence challenges that were both general (e.g., side effects) and context-dependent (e.g., not wanting to use PrEP while breastfeeding); 40% of these women discontinued PrEP due to these challenges (Seidman et al., 2016).

Potential side effects were a significant concern for trans women as well (Fisher et al., 2017; Galindo et al., 2012; Sevelius et al., 2016b; Thomann, Grosso, Zapata, & Chiasson, 2018; Wood et al., 2017). Other barriers to PrEP among trans women include not wanting to add additional medications to their existing health regimen, logistical concerns about getting to PrEP-related appointments, PrEP-related stigma, and wanting more education about PrEP (Fisher et al., 2017; Sevelius et al., 2016b).

### Behavioral Risk

Findings related to risk compensation indicated that cis women do not regard PrEP as a replacement for other safer sex practices. In multiple studies, cis women regarded PrEP as a back-up plan for condom failure or as an extra layer of protection (Auerbach et al., 2012; Collier et al., 2017; Park et al., 2017). Younger cis women endorsed this view of PrEP more so than older women (Rubtsova, Wingood, Dunkle, Camp, & DiClemente, 2013). Studies with mixed-cisgender samples found that participants did not believe PrEP to have an effect on condom use (Smith et al., 2012) and, indeed, that PrEP use was not associated with a decrease in condom use among cisgender women, regardless of their race or ethnicity (Flash et al., 2016). Trans women, too, expressed fear that disinhibition due to PrEP would result in less condom use (Galindo et al., 2012). Two studies documented increased risk, however, in contrast to the findings in cis women; a decrease in condomless receptive anal intercourse was less likely to occur among trans women compared with cis men after initiating PrEP (Marcus et al., 2013).

## Interpersonal Level Findings

### Sexual and Relationship Partners

The most common indicator for PrEP use among cis women in the U.S. was being in a serodiscordant partnership (Bien, Patel, Blackstock, & Felsen, 2017; Blackstock et al., 2017). Cis women in serodiscordant couples had generally positive feelings about PrEP, as it allowed them to remain HIV negative while maintaining their intimate relationships (Bazzi, Leech, Biancarelli, Sullivan, & Drainoni, 2017; Mahoney, Weber, Bien, & Saba, 2015; Park et al., 2017).

In multiple studies, cis women reported that taking PrEP would make their partners upset, mistrustful, and suspicious of infidelity (Auerbach et al., 2015; Goparaju et al., 2017). However, these concerns may not depress actual use of PrEP (Goparaju et al., 2017). Mistrust of men was reported as a facilitator of PrEP use (Auerbach et al., 2012). A study of black cis women found that a benefit of PrEP is that it can be a woman-controlled method of HIV prevention, but participants in this study were also concerned that PrEP may encourage remaining in unhealthy relationships and sex with “risky” partners (Bond & Gunn, 2016). Notably, these were perceived outcomes, not reported outcomes. Characteristics of women’s sexual and relationship repertoires are also associated with PrEP. Among younger, primarily black, cis women, those who had concurrent partners in the past year were more likely to report potential PrEP adherence than those without (Rubtsova et al., 2013) and cis women who have sex with men were more likely to initiate PrEP while in a serodiscordant relationship than were cisgender women who have sex with women and men (Garner, Wilson, Hirsch, Skalweit & Van Epps, 2016).

Proceptive intentions were believed to facilitate PrEP use among women in serodiscordant partnerships by reducing risk of HIV transmission while trying to conceive (Collier et al., 2017). One study found that aspirational notions of “normal” relationships and families contributed to women’s fertility desires and PrEP offered these women safer conception through condomless sex, which was preferred over assisted reproductive methods (Bazzi et al., 2017). The role of pregnancy as it relates to PrEP is unique to cisgender women. One study estimated that perhaps 10% of pregnant women in urban areas are eligible for PrEP and these women were more likely to be younger, African-American, without a partner and with lower educational attainment (Fruhauf & Coleman, 2017).

Intimate partner violence (IPV) differentially influenced PrEP willingness and acceptability in cisgender women. In one study, young black cis women with a history of IPV were more willing to use PrEP than those with no history of IPV (Willie et al., 2017). This finding may also be influenced by race, as a similar study with fewer black women in the sample found that those with a history of IPV demonstrated less PrEP acceptability than those without (Garfinkel et al., 2017). Among cis women who did sex work, client-perpetrated violence was associated with greater interest in PrEP (Peitzmeier et al., 2017).

Interpersonal stigma was a concern for trans women when considering PrEP; they did not want to be perceived as promiscuous or high-risk by sexual and/or romantic partners (Biello et al., 2018). Trans women who engaged in sex work reported that PrEP allowed them to protect themselves from HIV in situations where they had limited power to negotiate condom use (Sevelius et al., 2016b).

### Family and Friends

Gender was a significant factor in perceived interpersonal stigma associated with PrEP. Cis women were less concerned than men that others would find out they were taking PrEP (Koper et al., 2015). Nevertheless, anticipated stigma from friends and families was a barrier to PrEP uptake for cis women, who were worried about being judged as promiscuous, or assumed to be either HIV positive or in a serodiscordant partnership (Bazzi et al., 2017; Goparaju et al., 2017; Park et al., 2017; Smith et al., 2012).

Subjective norm influenced PrEP intention and use particularly among young, primarily black women, who had greater intention of PrEP use if they believed their friends would also use PrEP (Dunkle et al., 2008; Rubtsova et al., 2013). Social norms differentially influenced actual PrEP use by race, as black women were more likely than white women to use PrEP if their friends did too (Wingood et al., 2013).

For trans women, friends and social media were common sources of PrEP awareness, and social networks were also key determinants of PrEP uptake (Wood et al., 2017). Among a sample of young transgender people that included trans women, a parent or guardian’s acceptance functioned as either a barrier or a facilitator for PrEP use, depending on the adult’s level of acceptance (Fisher, Arbeit, Dumont, Macapagal, & Mustanski, 2016; Fisher et al., 2017).

## Community Level Findings

Extant findings at the community level primarily concerned the healthcare infrastructure present in one’s community. For example, one facilitator of PrEP among a sample that included young black cis women was the availability of conveniently located pharmacies where they could access medication (Smith et al., 2012). While some were willing to access medication at hospitals or clinics, overall, the preference was for locations that were familiar and convenient for those without cars or limited funds for gas and relied on public transit (Smith et al., 2012). Additionally, the presence of syringe exchange programs (SEPs) in the community facilitated PrEP awareness among WWID. Those WWID who received HIV prevention information at an SEP were seven and a half times more likely to be aware of PrEP than women who did not (Walters et al., 2017a, b). PrEP knowledge among staff at these community resources can act as a barrier. One study found that social service providers operated under the misconception that PrEP is indicated only for MSM (Collier et al., 2017). Available health resources in one’s geographical community facilitated PrEP uptake among trans women. Specifically, clinics that offer trans-specific services (e.g., hormone therapy) from trans-competent clinicians (Sevelius et al., 2016b) facilitated PrEP use.

Community-level stigma related to being recognized by those working where PrEP would be accessed functioned as a barrier to PrEP among black cis women (Bond & Gunn, 2016). Among both cis and trans women, PrEP was not only regarded as an individual-level prevention strategy, but as a community-level intervention, with the potential to lower incidence in their communities especially among the at-risk populations therein (Auerbach et al., 2015; Collier et al., 2017). Among young trans women, lower collective self-esteem was related to increased odds of PrEP indications (Kuhns et al., 2016), and trans women perceived little community outreach about PrEP, believing MSM to be the targets of those messages to the exclusion of other at-risk groups (Biello et al., 2018).

## Institutional/Organization Level Findings

### Healthcare Providers and Medicine

The knowledge and beliefs of healthcare providers (HCP) influenced PrEP awareness, intention, and uptake among cis and trans women. PrEP use was highest in those cis women whose HCP recommended it (Dunkle et al., 2008). However, communicating with HCPs about sex in general, and PrEP in particular, was a barrier, as women anticipated judgment from their providers (Auerbach et al., 2015; Goparaju et al., 2017; Okoro & Whitson, 2017). Anticipated stigma from providers prevented trans women from requesting PrEP and experienced stigma in health care related to gender inhibited trans women’s engagement and retention in care (Reisner et al., 2017; Sevelius et al., 2016b). Willingness to engage one’s HCP in a conversation about PrEP differed by age and race in cis women. Black cis women were less embarrassed to ask their HCP for PrEP than were white women, and younger white women were less embarrassed than were older white women (Wingood et al., 2013). Cis women reported that short health care visits prevent the development of the relationships necessary to discuss sexual health and behaviors with providers, including the need for PrEP (Goparaju et al., 2017), though they describe primary care and Ob/GYN providers clinics as the best people to educate women on, and deliver, PrEP (Auerbach et al., 2015).

Among cis women, an additional concern was clinicians lacking knowledge about PrEP, making patients responsible for educating providers (Auerbach et al., 2015). And, while some providers may feel positively about PrEP and have a basic working knowledge of PrEP, one study found that very few (10%) were familiar with clinical practice guidelines on PrEP (Shrestha et al., 2017c). Providers themselves have expressed the need for education on PrEP clinical guidelines to feel more confident prescribing PrEP to women (Finocchario-Kessler et al., 2016). Provider characteristics, such as knowledge of PrEP, older age, and the belief that PrEP empowers women were more willing to prescribe PrEP (Tripathi, Ogbuanu, Monger, Gibson, & Duffus, 2012).

Hesitancy to prescribe PrEP among clinicians was a barrier for cis and trans women. In general, this is related to the belief that it will result in risk compensation (Tripathi et al., 2012). Beyond indications, patient characteristics influence clinician’s decision-making around PrEP. In one study, providers less frequently intended to prescribe PrEP to “heterosexual patients with partners of unknown status” (a risk category of mainly cis women) than to MSM, TGW, and serodiscordant couples, though it is unknown if this difference was significant (Mullins et al., 2017). This same study found that prescribers were more comfortable providing PrEP to adult versus adolescent cis and trans women (Mullins et al., 2017). In a qualitative study of 30 racially diverse trans women, none reported ever having PrEP mentioned or offered to them by a medical provider (Sevelius et al., 2016b).

Studies have documented medical mistrust among both cis and trans women of color, which reduces PrEP interest and uptake (Galindo et al., 2012). Mistrust included concern about the applicability of PrEP research to black women given their underrepresentation in research studies, skepticism of pharmaceutical companies’ intentions in providing PrEP, anticipating stigma from within the healthcare system if taking PrEP, believing that the CDC cannot be trusted to provide accurate information on PrEP, and fear of purposeful infection with HIV (Auerbach et al., 2015; Bond & Gunn, 2016; Flash et al., 2014a, b).

### Schools, Churches, and the Media

Cis women voiced concern around the lack of funding for organizations/institutions that could provide education and administration of PrEP, believing that schools were one of the best places to provide women with information about PrEP, whereas churches were seen as barriers to uptake (Auerbach et al., 2012). This was the case among serodiscordant couples who received social support from their church communities which was not thought to extend to issues relating to HIV (Bazzi et al., 2017). Women in serodiscordant couples stressed the necessity of media strategies to increase PrEP awareness. These women felt that media strategies could normalize serodiscordant relationships, but that the media never publicized PrEP for serodiscordant couples who were not MSM (Bazzi et al., 2017).

## Policy/Societal Level Findings

### Social Stigma

Just as it was related to PrEP at the lower ecological levels, so too was stigma understood as a larger social force associated with PrEP. Black women in particular believed that the lack of dissemination of information about PrEP to them was influenced by the societal devaluation black people in the U.S. (Auerbach et al., 2015). Stigma was found to influence formulation preference among trans women who believed that injectable PrEP, because it is less visible than daily pills, could reduce stigma-related social harm (Biello et al., 2018). Despite feeling ignored by most PrEP outreach, one study found that trans women described PrEP being marketed to them in a way that perpetuates the idea that trans women “vectors” of HIV (Sevelius et al., 2016a, b).

### Economic and Health-Related Policies

The cost of PrEP was a well-documented barrier among trans women (Galindo et al., 2012) and cis women, regardless of race (Auerbach et al., 2012; Wingood et al., 2013), including those in serodiscordant relationships (Tripathi, Whiteside, & Duffus, 2013). Insurance and Medicaid coverage of PrEP were regarded as facilitators to PrEP use among cis women (Collier et al., 2017). Relatedly, concerns about insurance and Medicaid coverage along with the price of insurance coverage and copays depressed interest, uptake, and adherence (Auerbach et al., 2015; Goparaju et al., 2017; Seidman et al., 2016; Smith et al., 2012). The comparative affordability and accessibility of condoms compared to PrEP decreased preference for PrEP (Goparaju et al., 2015). The structure of health insurance was a barrier for healthcare providers as funding and reimbursement structures make it difficult to see and treat couples and monitor the uninfected partner in a serodiscordant couple (Finocchario-Kessler et al., 2016). The economic marginalization of trans women, resulting from the disparity in employment and income between transgender people and the general population, exacerbates the cost barrier to PrEP (Galindo et al., 2012). Economic marginalization can also increase the need for PrEP. For example, trans women who engage in sex work can earn more money for condomless sex, which thus provides a financial incentive for the higher-risk behavior that PrEP could make safer (Sevelius et al., 2016b).

## Discussion

Through a socioecological approach, this review attempts to distinguish relevant characteristics of individual women, their interpersonal relationships, and the community, institutional and larger social contexts of the U.S. in which they live, to elucidate findings that are specific to gender and not simply HIV transmission risk category. It further attempts to untangle individuals from the classification groups that may make it difficult to identify potentially important factors associated with PrEP use among cis and trans women.

Southern states accounted for more than half of new HIV diagnoses in the U.S. in 2017 (CDC, 2017) and HIV infection rates among women are highest in the Southern U.S. (CDC, 2019). Given this geographical disparity, it is of interest that the majority of study locations providing data on PrEP in cis and trans women (Table 1) are not the U.S. south. Coupled with a recent finding that the number of female PrEP users per new HIV diagnoses was lowest across all the southern states (Siegler et al., 2018), this points to the need for additional research related to PrEP alongside concerted efforts to improve access to PrEP among women in the southern U.S.

The findings we document here demonstrate that cis and trans women are willing to take PrEP once they know about it and find PrEP acceptable. However, awareness of PrEP was generally lower in cis and trans women when compared with cisgender men. The social marketing of PrEP as a medication for MSM may depress access and use by other at-risk groups including trans and cis women who at the individual level may not believe PrEP to be a medication for them. The implications here include the necessity of higher-level interventions meant to adjust and expand health promotion and public health messaging for PrEP to these specific groups of women.

Relatedly, the hesitancy among clinicians to prescribe PrEP to women should be addressed through enhanced medical education and training. The observed variations related to age and race in willingness to talk to an HCP about PrEP have important implications given that PrEP use is highest in women to whom it is recommended by their HCP (Dunkle et al., 2008). This further illustrates the importance of broad provider education about PrEP as an institutional-level intervention. Such an intervention should focus on ensuring that providers are not merely aware of PrEP or knowledgeable about who PrEP is appropriate for, but comfortable initiating conversations about and prescribing PrEP. This should cross almost all specialties and include nurse practitioners and physician assistants, as prescribing PrEP is not limited to physicians or those clinicians working in the areas of HIV or infectious disease.

Though many findings spanned multiple ecological levels, the significant focus on intra- and interpersonal factors associated with PrEP, as documented in this review, obscures a comprehensive understanding of the influences of higher-level forces on PrEP use. For example, understanding economic barriers to health care and medical mistrust among cis and trans women of color is necessary to understanding and improving their use of PrEP and HIV outcomes. Interventions aimed at increasing awareness or interest in PrEP have limited utility if the medications remain beyond women’s financial means or are only accessible through significant engagement with the healthcare system, and if institutional actors within the healthcare system are not comfortable discussing or prescribing PrEP.

We found a striking, persistent conflation of sex with gender and sexuality (Table 2). Sex, gender, and sexuality are all necessarily of interest to PrEP researchers. The lack of conceptual distinction between the three in the existing body of research prohibits a nuanced, clear understanding of how each differentially influences HIV exposure, acquisition, and prevention (Krieger, 2003). While there may be shared anatomical structures and behavioral repertoires between cisgender men and transgender women, trans women’s gendered lives differentially structure their HIV risk as well as their access to HIV prevention services (Grant et al., 2016). We echo the policy recommendation of the Center of Excellence for Transgender Health (Sausa et al., 2009) in calling for, at minimum, a two-question method for assessing gender identity and assigned sex at birth. Doing so can help reduce misclassification of participants. Beyond that, it is imperative that those in the field of HIV research be mindful of the related—but not entirely overlapping—roles of sex, gender, and sexuality in the conceptualization and design of studies as well as in the reporting of results. When HIV researchers include trans women in their studies, they should do so meaningfully, clearly and specifically reporting on their experiences (Grant et al., 2016).

We documented many similarities between cis and trans women, including lower awareness, uptake, and adherence compared to cis men. Lower adherence in cis women has different implications for PrEP efficacy than it does in trans women, given that PrEP becomes ineffective for cis women after fewer missed doses compared to men and trans women resulting from the lower bioavailability in vaginal mucosa compared to anal mucosa (Cottrell et al., 2016). Additional similarities between cis and trans women include concerns about side effects, fear of stigma, including healthcare stigma, medical mistrust, and the cost of PrEP as a barrier. Drawing parallels between cis and trans women as they relate to PrEP, as we have done here, can aid in conceptualizing trans women as women and not as MSM. In the context of HIV prevention interventions, the goal should be to make the same intervention available to as many people available based on evidence that it is effective in different gender groups. The Descovy controversy created an advocacy outcry because of the exclusion of cisgender women and illustrates a persisting problem with exclusion of cisgender women from clinical trials. This problem also extends to exclusion of transgender women from clinical trials. It is critical that HIV pharmaceutical prevention strategies are studied on cis and transgender women so that they are included in eventual approvals for their use.

It is equally important to recognize the differences between cisgender and transgender women as they relate to PrEP. For example, trans women’s concerns about remaining on hormone therapy and the need for trans-specific or gender-affirming care, are not shared by cis women. The impact of pregnancy on PrEP screening, uptake, and adherence are issues for cis women but not for trans women. One clear difference between cis and trans women was related to risk compensation, specifically condom discontinuation or nonuse during receptive anal intercourse among trans women (Marcus et al., 2013), with no corollary documented in the literature in cis women. It is necessary to incorporate understanding of these divergences into public health and prevention messaging and into targeted approaches within the field of HIV prevention. This is often done with messaging for MSM versus men who have sex with women. A “one prevention message fits all” approach will not maximize opportunities to prevent new HIV infection in cis and transgender women.

Racial/ethnic differences feature prominently across socioecological levels. Race is related to risk perception in cis women with some evidence that despite being at high risk, minority women can perceive themselves to be at low or no risk from HIV infection (Khawcharoenporn et al., 2012). Compared to white women, black women had higher PrEP willingness, greater PrEP intentions, a more positive subjective norm for PrEP use, and were less likely to be embarrassed asking healthcare providers for PrEP. Medical mistrust was a barrier to care unique to cis and trans women of color. The differing barriers and facilitators to healthcare access in general and PrEP access in particular must be considered in targeted efforts to increase women’s access to PrEP. PrEP health promotion and messaging should additionally be culturally appropriate, and depending on geographical location, multilingual options may be necessary.

Stigma features prominently as a barrier to PrEP across multiple socioecological levels as well. Online telemedicine options such as plushcare.com and Nurx.com, currently expanding in the U.S., may not reduce this stigma, but have the potential to reduce exposure to stigma (Chapman, 2017; Knight, 2019). Accessing PrEP via these options may alleviate anticipated stigma and judgment from a healthcare provider given that the person prescribing PrEP via telemedicine is not the patient’s primary care provider in their medical home. Telemedicine may also reduce exposure to stigma as individuals accessing PrEP through telemedicine do not have to sit in a physical PrEP clinic waiting room, many of which serve primarily MSM and may not feel welcoming to trans women or be considerate of their needs (Escudero et al., 2015). Outside of stigma, telemedicine can facilitate engagement in care where the infrastructure to support the PrEP care continuum is lacking.

States with the highest number of uninsured individuals are also those with the highest number of overall HIV incidence rates (CDC, 2019). For women in the studies reviewed, insurance coverage and cost of PrEP factored into their interest and uptake. Pharmaceutical assistance programs provided by drug manufacturers may help address these issues, but both prescribers and patients may be unaware of the extent to which this assistance is available. Telemedicine may also reduce costs associated with an in-person provider visit including travel costs, as one needs only a computer or mobile device which are increasingly common even among those in lower-income brackets (Gonzales, 2015).

There are some limitations to this review which should be considered. The current CDC classification system, which itself conflates sex (i.e., male and female), gender (e.g., men, women, trans women) sexuality (e.g., heterosexual and gay), and behavior (e.g., “sex with men”), was reproduced throughout the literature reviewed (CDC, 2018). Researchers’ lack of distinction between sex and gender terms occasionally made it difficult to definitively determine who was represented in the studies reviewed. For example, a study sample may have been described alternately as “female” and as “women” throughout the research report. In such cases, we do not know whether the sample included only cis women, both cis and trans women, or whether trans men were included and misclassified. Despite sex and/or gender frequently being inclusion or exclusion criteria, few studies described how data on sex and/or gender were collected. Given the conventions of language in biomedical research, we assumed that the researchers were referring to cisgender men and women when they used the terms men and women or male and female, unless otherwise specified in the methods or discussion. This may have led to misclassification in the current review when the sex and/or gender of the study sample was not made clear in the original research article or abstract.

Another limitation of the present review is the utilization of one database, PubMed. This limitation was mitigated by searching the abstract databases for the three major HIV conferences indicated in the methods section, though we could have strengthened this further by reviewing abstracts submitted to other pertinent conferences, for example the American Public Health Association Annual Meeting. We could have further strengthened this review by conducting a bibliography review to minimize the risk of excluding potentially important literature. Given the advancements made in PrEP research over the past decade, some of the more recent findings included in this review are more relevant than earlier findings. This review includes literature published before 2018, and the need for continued research on PrEP in women is critical, especially when structural barriers to PrEP continue, for example, the lack of Descovy approval for all populations. A final limitation concerns generalizability both inside and outside the U.S. This review was limited to research that had at least one study site in the U.S., and the findings we present may not be applicable in other countries. Notably, most of the study locations in this review are in the geographical northeast of the country. The lack of literature with regard to PrEP from the south means that our findings may not be generalizable to the parts of the U.S. where the majority of people living with HIV are to be found and where, overall, the highest number of new HIV diagnoses occur.

Despite these limitations, this review represents a robust overview of the recent literature on PrEP in cis and trans women in the U.S. through a socioecological lens. Over 70% of the papers included herein were published or presented at conferences after the cutoff date for the most recently published review that included both cis and trans women (Koechlin et al., 2017). As such, we provide a timely updated review in this area. To our knowledge, this remains the most up-to-date review of PrEP in our populations of interest. One that allows for parallels to be drawn between cis and trans women and—crucially—distinguishes between MSM and TGW. We additionally echo authors of previous reviews on PrEP in transgender women in calling for sex- and gender-based analyses which recognize the different social and biological realities of individuals in the same risk group, which differentially structure their risk of exposure and limit their access to HIV prevention and care (Krieger, 2003; Young & Meyer, 2005).
